# Characterization of Key Odor-Active Compounds in Draft Beers for the Chinese Market Using Molecular Sensory Science Approaches

**DOI:** 10.3390/molecules29112537

**Published:** 2024-05-28

**Authors:** Yu Zhang, Sinuo Li, Qi Meng, Huanlu Song, Xiaojun Wang

**Affiliations:** 1 Laboratory of Molecular Sensory Science, School of Food and Health, Beijing Technology and Business University (BTBU), Beijing 100048, China; 2 College of Food Science and Technology, Yunnan Agricultural University, Kunming 650201, China

**Keywords:** draft beer, sensory evaluation, electronic nose, two-dimensional comprehensive gas chromatography–olfactometry–mass spectrometry analysis, aroma extraction dilution analysis, odor-active compound

## Abstract

Beer is a popular alcoholic beverage worldwide. However, limited research has been conducted on identifying key odor-active components in lager-type draft beers for the Chinese market. Therefore, this study aims to elucidate the odor characteristics of the four most popular draft beer brands through a sensory evaluation and an electronic nose. Subsequently, the four draft beers were analyzed through solid-phase microextraction and liquid–liquid extraction using a two-dimensional comprehensive gas chromatography–olfactometry–mass spectrometry analysis (GC×GC–O–MS). Fifty-five volatile odor compounds were detected through GC×GC–O–MS. Through an Aroma Extract Dilution Analysis, 22 key odor-active compounds with flavor dilution factors ≥ 16 were identified, with 11 compounds having odor activity values > one. An electronic nose analysis revealed significant disparities in the odor characteristics of the four samples, enabling their distinct identification. These findings help us to better understand the flavor characteristics of draft beer and the stylistic differences between different brands of products and provide a theoretical basis for objectively evaluating the quality differences between different brands of draft beer.

## 1. Introduction

Beer is a popular alcoholic beverage worldwide [1]. It is produced by fermenting materials such as malt, water, and hops. It is also known as “liquid bread”, owing to its abundance of essential amino acids, vitamins, and nutrients required by the human body [2]. Beer can be classified as draft, fresh, or pasteurized, depending on whether it is sterilized or not [3]. Among them, draft beer is more popular among consumers because it remains unsterilized by heat, offering a fresh and mild flavor [4].

Draft beer is brewed through a specialized process that includes rigorous microbiological control and a three-stage filtration, including a 0.45-micron microporous filtration. Significantly, draft beer is not pasteurized or instantly autoclaved and has a high degree of biostability [4]. As is common knowledge, distinct flavor profiles exist among various beer types, such as draft, fresh, and pasteurized, making them readily distinguishable to the discerning palate. It is not easy to distinguish between beers of the same category, but differences in yeast strains and fermentation processes can lead to nuances in flavor. Typically, only experienced and loyal consumers or professional tasters can detect these nuances [5]. Consequently, determining the major flavor characteristics of various brands of draft beer is critical. This not only assists consumers in selecting the most suitable brand of draft beer but also supports food quality control and enhances the flavor quality of draft beer.

An important sensory aspect of beer is its aroma, which is predominantly influenced by the volatile components within the beverage [6]. For alcoholic beverages, gas chromatography (GC) combined with sensory analysis is usually used to detect the odor-active compounds it contains. Currently, common sample preparation techniques used to analyze alcoholic beverages include dynamic headspace (DHS), liquid–liquid extraction (LLE), multiple stir absorptive extraction (mSBSE), solid-phase extraction (SPE), and solid-phase microextraction (SPME). Each extraction method has its own set of advantages and disadvantages [7,8]. The extraction of all the key odor-active compounds can be achieved by utilizing a combination of these methods [9]. In a comparison of different sample preparation techniques, Zhang et al. found that SPME is suitable for the collection and concentration of hydrocarbons (including terpenes), esters, and alcohols. DHS may not be the ideal technique for the analysis of volatile organic compounds (VOCs) in beer, and mSBSE measured fewer hydrocarbons in mixed beers than SPME did. In addition, they mentioned that one of the main drawbacks to the use of SPE for the analysis of VOCs in beverages is the Cumbersome extraction procedure [10]. Owing to its flexibility and selectivity, the headspace solid-phase microextraction (HS-SPME) technique is often used to analyze volatile compounds in beer and other liquors [11]. In addition, LLE is a common extraction method mostly used to analyze volatile compounds in alcoholic beverages; it offers the advantages of straightforward sample manipulation and cost-effectiveness [12]. SPME is used to concentrate low-boiling compounds in beer, whereas LLE is utilized to concentrate medium-to-high-boiling compounds. Consequently, this study combines both LLE and SPME as pretreatments to ensure a comprehensive extraction of the volatile compounds from the draft beer.

It was found that headspace solid-phase microextraction combined with gas chromatography–mass spectrometry (HS-SPME–GC–MS) has been successful in measuring a wide range of volatiles in beer, such as organic acids, carbonyl compounds, esters, alcohols, fatty acids, and monophenols [7]. De Lima et al. characterized 34 volatile compounds in lager beer, including esters, alcohols, acids, and ketones, using HS-SPME–GC–MS [13]. Riu-Aumatell et al. detected 59 volatile constituents from beer, including 20 alcohols, 13 esters, and eight aldehydes, using HS-SPME–GC–MS [14]. Zhou et al. used LLE–GC–MS to analyze the differences in the flavor compounds among five Baiyunbian-aged liquors [15]. Thompson-Witrick et al. recognized 36 volatile compounds in Lambic beers [16]. They used continuous liquid–liquid extraction/solvent-assisted flavor evaporation (CLLE/SAFE) for this purpose. Additionally, they compared the effectiveness of CLLE/SAFE with SPME for extracting volatile compounds from Lambic beers. The results indicated that CLLE/SAFE was efficient in extracting acids, while SPME excelled in isolating esters. A study has shown that the differences in flavor metabolites between the different brands of draft beers were not significant, but the differences in the sensory evaluation analysis were significant [4]. However, there are no reports on studies using molecular sensory science approaches to explore the flavor metabolites responsible for the differences in the sensory evaluation of different brands of beer.

Therefore, the aims of this study were to (1) elucidate the odor profile of four draft beers through a sensory evaluation and the utilization of an electronic nose (E-nose); (2) characterize odor-active compounds in draft beer using SPME and LLE in combination with a two-dimensional comprehensive gas chromatography–olfactometry–mass spectrometry analysis (GC×GC–O–MS); (3) identify and quantify key odor-active compounds using molecular sensory science approaches; (4) explore the correlation between key odor-active compounds in the draft beers and a sensory evaluation; (5) construct a recombination model for the odor profile of draft beer. This study offers valuable insights into the odor characteristics of mainstream draft beers in the Chinese market. Meanwhile, it preliminarily revealed the odor-active compounds responsible for the differences in the sensory evaluation of different brands of beers and provides a theoretical basis for objectively evaluating and improving the quality of different brands of draft beer.

## 2. Results and Discussion

### 2.1. Analysis of E-Nose

**Figure 1:**
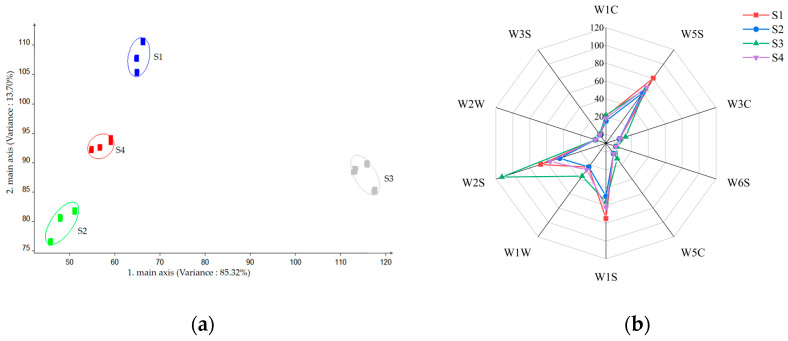
Sensors response (W1C, W5S, W3C, W6S, W5C, W1S, W1W, W2S, W2W, W3S) from the electronic nose (E-nose) analysis of the samples Snowflake (S1), Tsingtao (S2), Budweiser (S3) and Yanjing (S4), through PCA scores plot (**a**) and radar chart (**b**).

Figure 1a presents the steady-state response values of the 10 sensors of the E-nose, which were selected as the eigenvalues for the principal component analysis (PCA). The contribution of variance for the first principal component (PC1) was 85.32%. The second principal component (PC2) was 13.70%. Their cumulative variance contribution was 99.02%, which indicated that PC1 and PC2 represented the volatile profile of beers [17]. The PCA results showed that the four beers differed significantly in terms of PC1, with no overlap. This indicates that the beer samples have sensory differences and can be distinguished from each other. S3 was further separated from the remaining three samples in PC1, suggesting that the volatile profile of this beer differed significantly from that of the remaining three. Furthermore, the greater separation observed between S1 and S2 in PC1 suggests that the olfactory distinction between S1 and S2 is more pronounced. The smaller distances between S1 and S4 and between S2 and S4 in PC1 indicated that there was some similarity in the volatile profile of the two groups.

The 10 sensors, which displayed varying responses to the samples (Figure 1b), exhibited distinct characteristics. Significantly, the three sensors with the highest correlations, namely W5S, W2S, and W1S, corresponded to most alcohols, aldehydes, ketones, and broad-range methane. It is evident that corresponding to most alcohols, aldehydes, ketones, and broad-range methane mainly caused the different volatile profiles of the four beers. Although E-nose can distinguish differences in sample odors, it is unable to characterize the odor profile of the samples or identify odor-active compounds at the molecular level. Therefore, a molecular sensory science approach was used to identify the main odor-active compounds of the draft beers.

### 2.2. GC×GC–O–MS Analysis

**Figure 2:**
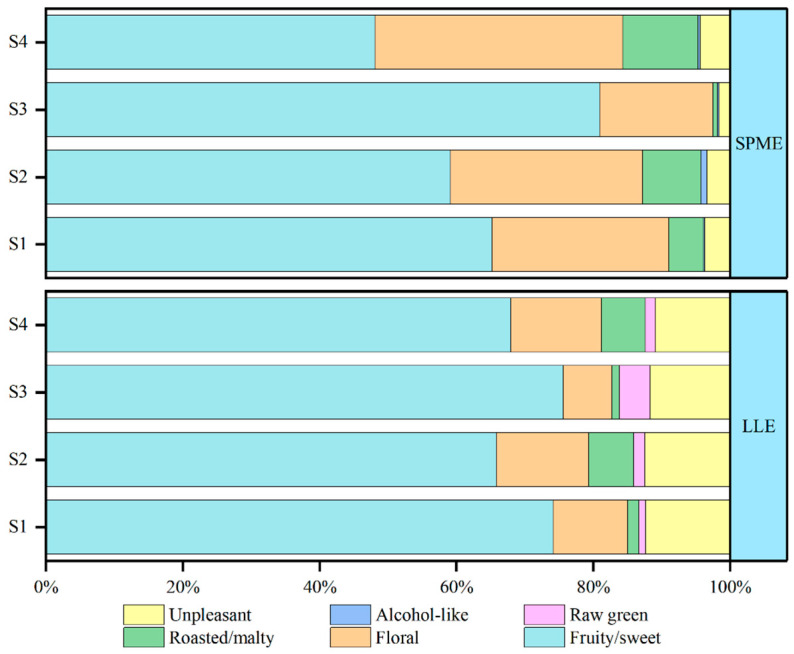
The percentage stack bar chart: the score of each odor descriptor compound accounts for the percentage of the total score of odor-active compound in draft beers S1–S4. The sum of FD factor of all compounds in each odor attribute was used as its intensity score.

Fifty-five odor-active compounds in draft beer were detected using GC×GC–O–MS (Table 1). Based on their odor characteristics, the compounds were classified as fruity/sweet, floral, roasted/malty, raw green, alcohol-like, and unpleasant odors; unpleasant odors included sour and some unacceptable odors. The interaction of the various odor characteristics resulted in the overall odor of the draft beer. The analysis of each odor profile revealed differences in the number of characteristic compounds per sample, corresponding to the different pretreatment methods. In both pretreatment results, the total number of odors was similar for S1, S2, and S4, with the lowest count observed in the case of S3. Furthermore, the odor intensities of the four samples differed significantly from high to low for S1, S4, S3, and S2. The degree of odors within the six odor attributes of each sample was similar, suggesting a comparable complexity of the odor-active compounds. In addition, a substantial variation in odor intensity percentages was observed among the samples (Table S2). From Figure 2, it can be seen that the odor differences among the four samples may be credited to fluctuations in the intensity of odor-active compounds with high FD factors. Fruity/sweet, floral, roasted/malty, and unpleasant odors were the most common among the four samples tested.

The category of fruity/sweet odors includes fruity, sweet, creamy, and caramel-like odors. Thirty-five odor compounds with fruity/sweet characteristics were detected in four samples, which included esters, alcohols, acids, ketones, and furans. Significantly, the higher alcohols are the most representative compounds in beer, and they are produced from amino acids, either by wort (Ehrlich mechanism) or by sugar metabolism (biosynthetic mechanism) [18]. Higher alcohols and acyl coenzyme A synthesize esters cannot be produced without the production of higher alcohol precursors [19]. Most esters give the beer a fruity flavor [15]. Several common compounds found in beer include isoamyl acetate, ethyl caproate, and ethyl caprylate, which are associated with banana, apple, and fruity flavors, respectively [20]. Furthermore, these compounds were detected in four beer samples. Lactones in beer are produced from malt, hops, and yeast metabolites of amino acids and hydroxylated fatty acid precursors. Although the concentration of lactones is below its threshold in beer, they can provide a fruity, sweet flavor in the beer by acting synergistically [21]. In four beers, we found gamma-Butyrolactone, which provides a caramel-like sweetness to the beer.

In addition, the beer samples contained four compounds with floral odor characteristics. Among these compounds, phenethyl alcohol, known for its rose-like aroma, is commonly found in lagers as a byproduct of alcoholic fermentation. Another compound with a similar rose-like scent is 2-phenylethyl acetate, which is a distinctive ester often associated with lager beer varieties.

A roasted/malty odor includes scents of roasted, malty, and nutty characteristics. Five roasted/malty flavor compounds, including alcohols, aldehydes, and ketones, were found in the beer samples. Among these samples, 3-methyl-1-butanol showed a strong malt flavor during the sniffing process and was the most common higher alcohol in beer species [22]. Furfural is a product of the Maillard reaction formed during malt baking and brings a nut-like flavor to beer [14]. Additionally, 2,3-pentanedione, a product of lipid oxidation, is a primary volatile component found in barley or malt. Both 2,3-pentanedione and benzaldehyde contribute to a nutty flavor in beer [23].

Six unpleasant compounds were identified, mainly consisting of acids, including four short-chain fatty acids, namely acetic, butyric, hexanoic, and isovaleric acids. Short-chain fatty acids, carbonyl compounds, and esters have been reported to be major volatile compounds in beer [24]. Acids are an important group of compounds that can influence the organoleptic properties of beer with fruity, cheesy, and fatty odors. They also contribute to bitterness, astringency, and sourness [25]. For example, acetic, butyric, and hexanoic acids are known for their sour flavor.

In addition to the above three odor attributes, a certain percentage of raw green flavor was present. The raw green odors included fresh and cucumber-like odors. In this study, the compounds with raw green odors were hexanal, octyl acetate, and decanal. Among them, hexanal and decanal are recognized as the primary volatile compounds found in barley, which serves as the raw material for beer production [26]. These two compounds are key odorants in barley and malt [27].

### 2.3. Identification of the Important Odor Compounds in Draft Beer

In this study, important odor compounds in the samples were identified using AEDA. Table 2 shows that there were 22 key odor compounds with FD factors ≥ 16. Among these odor compounds, esters were the most abundant, with ten compounds, followed by alcohols with six compounds, acids with four compounds, and the least abundant were ketones and aldehydes, with one compound each. This indicates that esters and alcohol compounds have a remarkable influence on the odor profiles of draft beer.

Esters are present in small concentrations; however, they have a vital function in determining the beer flavor owing to their low odor threshold [22]. Isoamyl acetate and 2-phenethyl acetate are essential esters in beer [28]. Ten esters were identified in beer; among them, isoamyl acetate had the highest FD factors of 32–256, ethyl caproate and ethyl butyrate had FD factors of 8–64, and 1-heptyl acetate and 2-phenylethyl acetate had the highest FD factors of 64. These short-chain fatty acid esters make a greater contribution to the beer flavor than longer-chain esters with lower volatility, while the short-chain fatty acid esters mentioned above bring a fruity and floral aroma to the beer [29].

In this study, we identified six key alcohol compounds, including 2-methyl-1-propanol, 1-butanol, 3-methyl-1-butanol, 1-hexanol, furfuryl alcohol, and phenethyl alcohol. Among these compounds, 2-methyl-1-propanol contributes a fruity flavor to the beer and has the highest FD factor, which ranged from 256–512, based on analytical results with LLE. However, it has a lower FD factor when SPME is used as a pretreatment. This variation in the FD factor can be attributed to the characteristics of the fiber extraction head. It appears to be more suitable for separating esters and less effective at isolating alcohols, acids, and other compounds owing to its polarities and proximity to specific compounds [16]. 1-Butanol and 1-hexanol impart apple and hawthorn flavors to the beer, respectively. 1-Hexanol had a higher FD factor of 8–64 with LLE, and the FD factor of 1-butanol ranged from 2 to 16. Additionally, 3-Methyl-1-butanol, which has a malty flavor, exhibited an FD factor of 8–64 using LLE. Phenethyl alcohol has a rose-like aroma, with an FD factor of 16–128 with LLE.

Studies have shown that the odor of beer is the result of various compounds, with alcohols, organic acids, and esters being the three most influential compounds [30]. Among the key compounds identified in this study, esters, alcohols, and acids were the top three. This finding confirmed the findings of Niu et al. Among the acidic compounds, acetic and isovaleric acids had the highest FD factors of 64, whereas butyric and hexanoic acids had lower FD factors. These acidic compounds assist in the formation of astringency in beer [16].

**Table 2:** The important odor-active compounds in four brands of draft beer *^1^*.

No.	Compound	Selected Ion *^2^* (*m*/*z*)	Standard Curve	R^2^	Odor Threshold (μg/L)	Concentration *^3^* ± SD *^4^* (μg/L) (OAVs *^5^*)			
						S1	S2	S3	S4
1	Ethyl propionate	102, 57, 75	y = 0.0556x	0.9832	140 *^6^*	<1 ^a^ (<1)	<1 ^d^ (<1)	<1 ^b^ (<1)	<1 ^c^ (<1)
2	Ethyl butyrate	101, 43, 71, 88	y = 0.0649x	0.9757	367 *^7^*	<1 ^b^ (<1)	<1 ^c^ (<1)	<1 ^a^ (<1)	<1 ^c^ (<1)
3	Ethyl isovalerate	115, 57, 85, 88	y = 0.0014x	0.9792	1.6 *^6^*	134.3 ± 1.4 ^a^ (84)	133.6 ± 1.9 ^a^ (83)	37.6 ± 1.6 ^d^ (24)	127.7 ± 0.5 ^c^ (80)
4	2-Methyl-1-propanol	74, 31, 41, 43	y = 0.0008x	0.9886	56.5 *^6^*	163.3 ± 4.5 ^a^ (3)	66.8 ± 0.6 ^c^ (1)	133.3 ± 2.7 ^b^ (2)	139.6 ± 6.4 ^b^ (2)
5	Isoamyl acetate	87, 43, 55, 70	y = 0.023x	0.9819	0.15 *^6^*	72.7 ± 3.1 ^a^ (485)	34.4 ± 1.5 ^c^ (229)	27.7 ± 1.3 ^d^ (184)	51.8 ± 3.7 ^b^ (345)
6	1-Butanol	56, 31, 41, 43	y = 0.0001x	0.9947	459.2 *^6^*	1752.2 ± 35.7 ^a^ (4)	757.0 ± 75.2 ^d^ (2)	1622.9 ± 69.7 ^b^ (4)	1346.1 ± 50.1 ^c^ (3)
7	Isoamyl propionate	101, 43, 57, 70	y = 0.0035x	0.9823	0.3 *^6^*	<1 ^c^ (<1)	<1 ^b^ (2)	1.2 ± 0.1 ^a^ (4)	<1 ^d^ (<1)
8	3-Methyl-1-butanol	70, 41, 42, 55	y = 0.0029x	0.9746	176 *^6^*	6519.9 ± 297.2 ^b^ (37)	6648.7 ± 393.0 ^b^ (38)	6057.4 ± 131.8 ^b^ (34)	14,832.1 ± 454.8 ^a^ (84)
9	Ethyl caproate	115, 43, 88, 99	y = 0.0434x	0.9785	1.2 *^6^*	5.4 ± 0.5 ^b^ (5)	2.8 ± 0.1 ^d^ (2)	7.1 ± 0.1 ^a^ (6)	3.7 ± 0.1 ^c^ (3)
10	1-Hexanol	69, 41, 43, 56	y = 0.0002x	0.9822	5.6 *^6^*	17.4 ± 0.5 ^bc^ (3)	23.2 ± 0.7 ^b^ (4)	151.7 ± 6.1 ^a^ (27)	13.9 ± 0.6 ^c^ (2)
11	3-Hydroxy-2-butanone	88, 43, 45	y = 0.0005x	0.9758	14 *^6^*	77.0 ± 1.7 ^c^ (6)	1184.8 ± 75.4 ^a^ (85)	47.0 ± 0.6 ^c^ (3)	159.4 ± 7.4 ^b^ (11)
12	Ethyl lactate	75, 29, 45	y = 0.0223x	0.9802	250,000 *^7^*	<1 ^a^ (<1)	<1 ^c^ (<1)	<1 ^c^ (<1)	<1 ^b^ (<1)
13	1-heptyl acetate	116, 43, 56, 70, 98	y = 0.0036x	0.9706	830 *^6^*	18.3 ± 0.9 ^a^ (<1)	7.1 ± 0.7 ^c^ (<1)	17.0 ± 0.6 ^a^ (<1)	12.7 ± 1.3 ^b^ (<1)
14	Acetic acid	60, 43, 45	y = 0.0024x	0.9786	200,000 *^7^*	402.5 ± 12.7 ^b^ (<1)	251.5 ± 14.6 ^c^ (<1)	74.9 ± 0.5 ^d^ (<1)	477.5 ± 17.2 ^a^ (<1)
15	Decanal	112, 43, 57, 70, 82	y = 0.0097x	0.9925	1.5 *^6^*	<1 ^d^ (<1)	<1 ^c^ (<1)	<1 ^a^ (<1)	<1 ^b^ (<1)
16	Butyric acid	73, 41, 60	y = 0.0073x	0.9789	1899 *^7^*	54.8 ± 3.5 ^a^ (<1)	41.1 ± 1.3 ^b^ (<1)	50.9 ± 3.2 ^a^ (<1)	35.9 ± 2.7 ^b^ (<1)
17	Furfuryl alcohol	98, 97, 81, 53, 41	y = 0.0031x	0.9766	282 *^6^*	387.0 ± 12.1 ^a^ (1)	282.4 ± 13.4 ^b^ (1)	359.2 ± 23.2 ^a^ (1)	233.3 ± 11.8 ^c^ (<1)
18	Ethyl caprate	155, 43, 88, 101	y = 0.1819x	0.9703	1500 *^8^*	1.0 ± 0.1 ^b^ (<1)	<1 ^d^ (<1)	1.2 ± 0.1 ^a^ (<1)	<1 ^c^ (<1)
19	Isovaleric acid	87, 43, 60, 69	y = 0.001x	0.9795	1230 *^7^*	314.5 ± 7.4 ^c^ (<1)	192.4 ± 4.2 ^d^ (<1)	563.1 ± 39.1 ^a^ (1)	396.2 ± 8.7 ^b^ (<1)
20	2-Phenylethyl acetate	104, 43, 91	y = 0.0925x	0.9696	19 *^6^*	30.8 ± 0.7 ^a^ (2)	5.3 ± 0.1 ^d^ (<1)	11.4 ± 0.7 ^c^ (<1)	21.1 ± 0.4 ^b^ (1)
21	Hexanoic acid	87, 41, 60, 73	y = 0.0024x	0.9718	8000 *^7^*	479.8 ± 7.4 ^a^ (<1)	283.2 ± 9.3 ^c^ (<1)	412.0 ± 9.8 ^b^ (<1)	413.1 ± 20.7 ^b^ (<1)
22	Phenethyl alcohol	122, 65, 91	y = 0.0021x	0.9726	140 *^6^*	2135.8 ± 99.8 ^b^ (15)	2139.1 ± 124.6 ^b^ (15)	1830.4 ± 58.4 ^c^ (13)	9118.6 ± 164.5 ^a^ (65)

*^1^* Draft beer of S1–S4. *^2^* The ions selected for quantitative analysis include mother ions and quantitative ions. The first ion in each cell is the quantitative ion. *^3^* Mean value in triplicate experiments, marking its significance with a–d. *^4^* SD, standard deviation. *^5^* Odor active value, calculated by dividing the concentration by the odor threshold in alcohol. *^6,7,8^* Odor threshold references are listed in the references section [31,32,33].

### 2.4. Quantification of Important Odor-Active Compounds and Odor Activity Value (OAV)

Twenty-two important odor-active compounds were quantified (Table 2). On the basis of the quantitative results, 1-butanol, 3-methyl-1-butanol, and phenethyl alcohol were found to be the primary odor-active compounds with high concentrations in the draft beer. In addition, the OAV of a compound is determined by a combination of its absolute concentration in the sample and the odor threshold, which can give us some idea of the importance of odor in the overall flavor [34]. Compounds with an OAV > one may affect the aroma presented by the draft beer to a certain extent. Based on the AEDA results, 22 important odor-active compounds were chosen to compute the OAV and validate the odor intensities associated with these essential odor compounds.

There were 11 odor-active compounds with an OAV > one in the four draft beers. Among these were five esters, alcohols, and one ketone. Among the esters, isoamyl acetate exhibited the highest OAV and FD factor, a finding that aligns with the assertion made by Liu et al., suggesting a positive correlation between the FD factor and the contribution of the odor compounds to the overall aroma [35]. Isoamyl acetate distinctly influenced the banana flavor of the beer, and its content in the four draft beers showed a significant variation, ranked in descending order as S1 > S4 > S2 > S3 (*p* < 0.05). In addition, ethyl isovalerate had the second-highest OAV after isoamyl acetate and contributed a fruity flavor to the beers. The content of this compound was higher and did not differ significantly between S1 and S2, and the lowest content was found in S3 (*p* < 0.05). Ethyl caproate also contributed more to the fruity flavor of the four draft beers. Significantly, there were differences in the contents of ethyl caproate among the four draft beers, with the order being S3 > S1 > S4 > S2 (*p* < 0.05).

Table 2 presents a list of all the five alcohols that had a relatively large effect on the odor of the draft beers. In all four beers, 3-hydroxy-2-butanone had an OAV > one, suggesting that this compound also affects the overall odor of the beer.

Among the key compounds identified in this study, some had high FD factors but low OAVs, mostly esters. The content of these esters in beer is low; however, they have a major influence on the beer odor, even at levels below their flavor thresholds, because of the synergistic effect between the esters [29]. Consequently, odor compounds with an OAV < one were equally important in the formation of a characteristic beer odor. The same ingredients were used in four draft beers; however, factors such as the proportions of ingredients used, the type of microorganisms, the period of fermentation, and temperature during the brewing process might have affected the development of these vital odor components.

### 2.5. Sensory Evaluation

**Figure 3:**
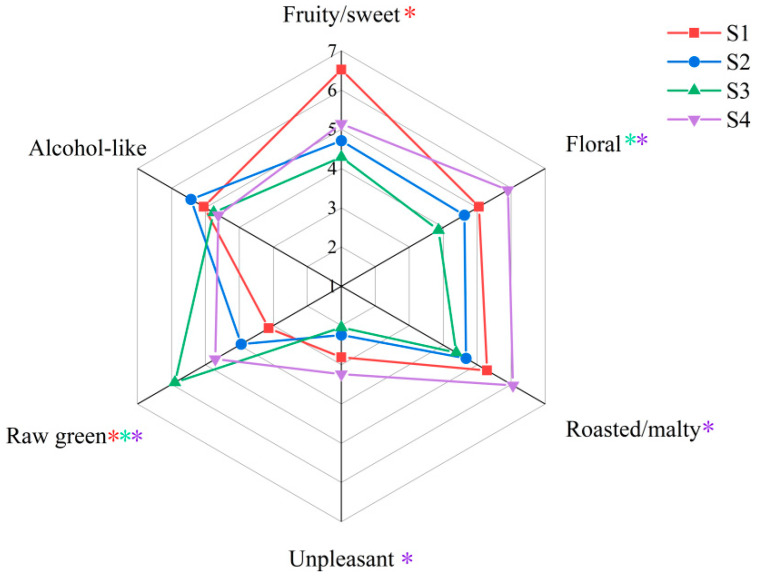
Sensory Evaluation of draft beer of S1–S4. * Odor descriptors with colored snowflake markers indicate significant differences (*p* < 0.05) in the sensory scores of samples of that color. One snowflake marker indicates a significant difference between the brand it represents and the remaining three. Two snowflake markers and above indicate that there is a significant difference between the brands they represent.

Figure 3 shows the results of the sensory evaluation of the four draft beers. The results suggested that the general odor of the draft beers was dominated by fruity/sweet, floral, and roasted/malty flavors. Among the four draft beers, the fruity/sweet odor garnered the highest scores, with S1 significantly different from the other three samples (*p* < 0.05), while S3 had the lowest score. This variation can be attributed to the substantial concentration of isoamyl acetate, the highest OAV compound associated with fruity flavor found in S1, and its lower presence in S3. A closer examination of Table 2 reveals that most compounds contributing to fruity/sweet flavors were more abundant in S1 than in the other three samples. The floral and roasted/malty flavors of S4 scored the highest among the four beers, which may be owing to the fact that the phenethyl alcohol compounds characterizing the floral flavors and the 3-methyl-1-butanol compounds characterizing the malty flavors had a higher OAV in S4 than in the other three samples.

Besides the three main odor characteristics mentioned above, four draft beers had raw green, alcohol-like, and unpleasant odors. The raw green odor of S3 was significantly higher (*p* < 0.05) than that of the other three samples, which may be attributed to the higher FD factor of decanal in S3. This divergence in sensory attributes between S3 and the other three samples aligns with the findings from the E-nose analysis. In this study, the quantity of odor-active compounds detected as raw green flavors in the beer was low. This may be due to the fact that some compounds with low FD factors were excluded from the scope of this study, resulting in some raw green odor active compounds not being detected. Considering the significance of the raw-green odor observed in S3, we intend to conduct more comprehensive investigations into this particular aspect in future research.

### 2.6. Correlation between Key Odor-Active Compounds in the Draft Beers and Sensory Evaluation

**Figure 4:**
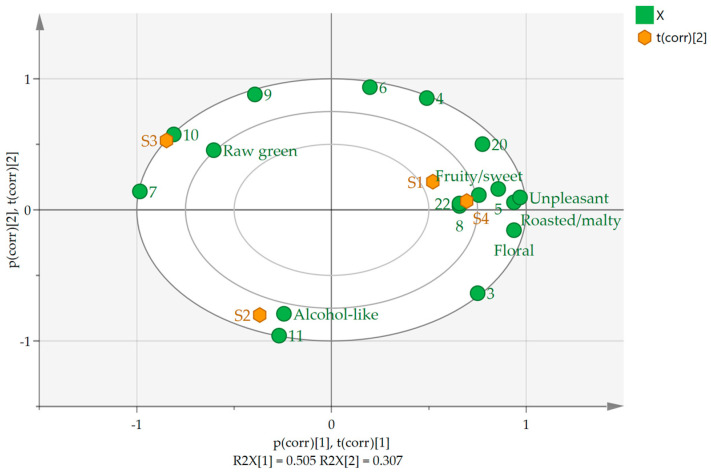
The PCA biplot of draft beer of S1–S4. Orange squares indicate the four draft beers, and green circles indicate the odor profiles of the draft beers as well as key odor compounds. Among them, individual key odor compounds were labeled in the order shown in Table 2: ethyl isovalerate (3), 2-methyl-1-propanol (4), isoamyl acetate (5), 1-butanol (6), isoamyl propionate (7), 3-methyl-1-butanol (8), ethyl caproate (9), 1-hexanol (10), 3-hydroxy-2-butanone (11), 2-phenylethyl acetate (20), and phenethyl alcohol (22).

The aim was to investigate what compounds caused sensory differences between the four brands and to examine the relationship between key aroma active compounds and sensory attributes in draft beer. A PCA analysis was performed based on the OAV values of the 11 key aroma active compounds and the olfactory scores of the sensory panelists in the draft beers. The PCA biplot obtained is shown in Figure 4. The fitting parameters of the first and second principal components were 50.5% and 30.7%, respectively, and the overall fitting parameter reached 81.2%, indicating that the model based on the OAV values of the key aroma active compounds and the olfactory scores of the sensory panelists could accurately characterize the samples.

In the PCA biplot, the greater the distance between the samples, the greater the difference between the samples, indicating that the differences between the samples were more pronounced. As shown in Figure 4, S1 and S4 were close to each other, which proves that their odor characteristics were similar, while the rest of the samples were at a greater distance, which proves that their odor characteristics were more different. Looking at the graph, it is easy to see that the fruity, floral, roasted, and unpleasant flavors were relatively close to S1 and S4, which could be the reason for the similarity of the odor characteristics of these two beers. The raw green flavor was closer to S3, and the alcohol flavor was closer to S2, which was also consistent with their sensory evaluations, indicating that the key aroma-active compounds in the draft beers were positively correlated with the sensory evaluations.

In addition, the correlation between sensory evaluation and key aroma active compounds in the samples was analyzed by the PCA. The results are shown in Figure 4; isoamyl acetate (5), 3-methyl-1-butanol (8), 2-phenylethyl acetate (20), and phenethyl alcohol (22) clustered at the right end of the graph were positively correlated with fruity/sweet, floral, roasted/malty, and unpleasant flavors. Similarly, 1-hexanol (10) was positively correlated with a raw green flavor, and 3-hydroxy-2-butanone (11) was positively correlated with an alcohol-like flavor. Antagonism and synergism might have influenced the volatility of these aroma compounds. They may be responsible for the fact that the odors presented by 1-hexanol and 3-hydroxy-2-butanone differed somewhat from the sniffing results.

### 2.7. Aroma Recombination

**Figure 5:**
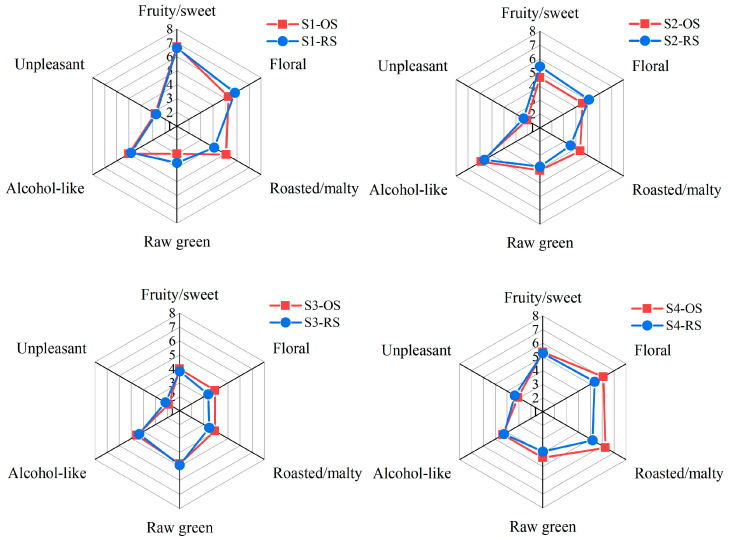
Sensory evaluation scores of S1–S4 (OS) and their odor reconstruction samples (RS).

Based on the quantitative findings, we combined 11 odor-active compounds that had an OAV > one in an alcoholic solution that matched the ethanol concentration and pH of the original beer. Sensory evaluation was performed on the reconstruction samples (RS) and original sample (OS). The results of the scores are shown in Table S3. Figure 5 presents the results. The distinction between the RS and OS of all the draft beers was not statistically significant (*p* > 0.05).

The RS of S1, S2, and S4 were characterized by dominant fruity/sweet, floral, and roasted/malty odors. Key contributors to these flavors were isoamyl acetate, phenethyl alcohol, and 3-methyl-1-butanol. Among these samples, S3 exhibited the highest score for its raw green flavor. However, it is worth noting that decanal, a compound responsible for the raw green flavor, had an OAV < one. This suggests that the raw green flavor in S3 may be the result of complex interactions among various odor compounds, leading to the unique sensory profile observed in the samples.

In conclusion, the odors of RS and OS are generally similar, but there are some differences. The differences between RS and OS arose from the inability to recombine certain volatile odor compounds with low FD factors, which play a significant role in the overall sensory experience. Nevertheless, the overall sensory evaluation scores indicated that the selected key odor compounds play an important role in contributing to the overall odor of draft beer.

## 3. Materials and Methods

### 3.1. Samples of Beer

According to the 2021 China Beer Brand Ranking, as published by Chnbrand, an authoritative brand-rating organization, the following top four beer brands were chosen: Snowflake (S1), Tsingtao (S2), Budweiser (S3), and Yanjing (S4). These four brands of lager draft beers were made in accordance with GB 4927-2008 guidelines (National Standard, 2008) [3]. The basic information of the samples is shown in Table 3. All samples were purchased online, and each brand belonged to its own same production batch. They were stored in a refrigerator at 4 °C.

The products of these four brands exhibit flavor differences due to the influence of raw materials, fermentation strains, and production conditions. However, Bai et al. proved that the product flavor of different brands has good consistency and stability by studying the consistency of different batches of draft beer produced by different factories within one year [4]. Therefore, the samples selected in this study can represent the product characteristics of each brand.

### 3.2. Chemicals

The standard chemicals involved in this study were obtained from Beijing Halfsia Technology Co., Ltd. (Beijing, China), and they were of high purity (99.0%) and chromatographic grade. These chemicals include ethyl propionate, ethyl butyrate, ethyl isovalerate, 1-butanol, isoamyl propionate, 3-methyl-1-butanol, ethyl caproate, 1-hexanol, 3-hydroxy-2-butanone, ethyl lactate, 1-heptyl acetate, acetic acid, decanal, butyric acid, isoamyl acetate, furfuryl alcohol, ethyl decanoate, isovaleric acid, 2-phenylethyl acetate, hexanoic acid, phenethyl alcohol, 2-methyl-1-propanol, 2-methyl-3-heptanone, and ethyl 2-ethylbutyrate. A series of alkane standards (C7-C30, 99%) were acquired from Sigma-Aldrich (St. Louis, MO, USA). Ultra-high-purity helium (99.999%) was acquired from Beijing He-Pu Beifen Gas Industry Co. (Beijing, China). Nitrogen (99.99%) was also from this company.

### 3.3. Analysis of E-Nose

An E-nose consists of a series of sensors and pattern recognition methods that are both convenient and inexpensive. This technology is widely applied in the field of food engineering [36]. The four draft beers were analyzed using PEN3 (Airsense, Schwerin, Germany). The PEN3 E-nose sensor consists of 10 metal oxide semiconductor (MOS) chemical sensing elements, each corresponding to a different main type of sensitive substance. The 10 MOS are W1C (mainly sensitive to aromatic components and benzene compounds), W5S (mainly sensitive to broad range compounds), W3C (mainly sensitive to ammonia and aromatic components), W6S (mainly sensitive to hydrocarbons), W5C (mainly sensitive to alkane, aromatics, and small polar compounds), W1S (broad range methane), W1W (mainly sensitive to inorganic sulfides), W2S (mainly sensitive to most alcohols, aldehydes, and ketones), W2W (mainly sensitive to aromatics and organic sulfides), and W3S (mainly sensitive to long-chain alkanes) [37]. An accurately weighed 10 mL sample of beer was poured into a 40 mL headspace bottle and settled for 30 min to ensure that the bottle was saturated with gas. Two syringe needles were used in this setup; they were fixed with polytetrafluoroethylene (PTFE) tubes and activated carbon filters, respectively. We made sure that both needles were inserted at the same time into the sealed headspace bottle containing the sample. During the assay, volatile gases were gradually absorbed from the sealed headspace of the vial and conveyed to the sensor at a controlled rate until the signal stabilized. The rate of flow of the sample was 300 mL/min, the flushing time was 100 s, and the measurement time was 200 s. To ensure the accuracy of the measurements, ten replicates were conducted for each of the four beer brands, effectively eliminating potential measurement errors.

### 3.4. Collection of Aroma Compounds from Samples

SPME: To extract aroma compounds from the beers, SPME was used. As previously stated [38] and modified, this involved placing 20 mL of beer into a headspace bottle. The volume of the headspace bottle was 40 mL. Samples were incubated in a water bath at 40 °C for 10 min. Subsequently, SPME sampling was performed using CAR-PDMS fibers (50/30 μm, Supelco, Bellefonte, PA, USA). Volatile odor compounds in the headspace vials were adsorbed using an extraction head for 30 min. The desorption phase was performed at 230 °C for 10 min, directing the compounds into the GC×GC–O–MS inlet for analysis. For every sample, it was analyzed three times.

LLE: Extraction and analysis of aroma concentrates of samples using the method mentioned by Zhai et al. [37]. At the same time, the method was optimized. A 50 mL portion of beer was extracted twice with dichloromethane (first with 10 mL and second with 15 mL). A magnetic stirrer was used to ensure that the extraction process was carried out efficiently. The speed was 1000 rpm, and the stirring time was 5 min each time. The sample obtained by partitioning was centrifuged, and the organic phase was collected. The conditions were set at 3500 rpm, the centrifugation temperature at 4 °C, and the centrifugation time at 10 min. The aqueous phase was removed with anhydrous sodium sulfate and then concentrated at 50 µL under a stream of nitrogen. Three parallels were made for each beer sample, and the resultant aroma concentrates were analyzed using GC×GC–O–MS.

### 3.5. Identification of the Odor-Active Compounds in Draft Beer through GC×GC–O–MS

The aroma compounds were analyzed using an Agilent GC–MS instrument (8890-5977B). In this setup, two columns were installed in the GC oven to separate the odorants, including a polar DB-WAX column (30 m × 0.25 mm × 0.25 μm, J&W Scientific, Folsom, CA, USA) and a medium-polar DB-17 ms column (1.85 m × 0.18 mm × 0.18 μm, J&W Scientific, Folsom, CA, USA). High-purity helium served as the carrier gas, flowing at a constant rate of 1 mL/min, and the inlet temperature was set at 230 °C in the splitless mode. The MS parameters involved ionization energy, with an ion source energy of 70 eV, an ion source temperature of 230 °C, a quadrupole temperature of 150 °C, and an *m*/*z* scan range of 40–400. A solid-state modulator SSM1800 (J&X Technologies, Folsom, CA, USA) was used to perform the heating and cooling processes between the two columns. The two-dimensional modulation period was set to 4 s.

For the SPME analysis, the oven temperature program started at 40 °C for the initial 5 min, up to 200 °C at 4 °C/min, and was then held at 200 °C for 8 min.

In the case of LLE, the oven temperature program began at 40 °C for the first 5 min, up to 230 °C at 4 °C/min, and held constant at 230 °C for 10 min.

GC×GC–O–MS was also equipped with an olfactory instrument (Sniffer 9100; Brechbuhler, Schlieren, Switzerland). The temperature at the sniffer port was set at 200 °C. Nitrogen was also used to introduce vapor through ultrapure water to prevent discomfort in the nasal passages. The ratio of sniffer port to the mass spectrometer is 1:1. Olfactory analyses were performed by three Chinese assessors (one male and two females, aged 25–35 years) who recorded the time and description of the odor characterization. Each assessor evaluated the samples three times.

The odor compounds were characterized by MS. Mass spectral peaks were checked against those in the NIST 17 library. Then, the identified compounds were validated by the linear retention indices (RI) of these compounds as well as by the results of odor description (O). The theoretical RI of the compounds is shown in Table S1. The RI of all odor compounds was determined by n-alkanes (C7–C30). The van den Dool and Kratz equation for RI is calculated as follows [39]:

RI = 100n + 100(t_a_ − t_n_)/(t_n+1_ − t_n_), (1)

Among them, t_a_: retention time of compound a in the sample; t_n_: retention time of the nth carbon atom n-alkane standard C_n_; the retention time t_a_ of sample a is bounded between the retention times of two neighboring n-alkanes C_n_ and C_n+1_.

### 3.6. AEDA

The main odor components of the draft beer were determined using AEDA. Odor-active compounds were analyzed at dilution by varying the split ratio during SPME sample handling. When the samples were processed using LLE, we diluted the odor concentrates of each beer in equal volumes with methylene chloride. Three experienced Chinese evaluators performed the AEDA. Confirmation of each odor required at least two of the three evaluators to sniff the odor. The extracted samples were thinned at a ratio of 2^n^ until no odor was detectable, at which point the dilution was at its maximum. The FD factor of every aromatic compound was interpreted as the maximum detectable dilution.

### 3.7. Quantitative Analysis

Based on the results of the AEDA, a more precise quantitative analysis of 22 major odorants with FD factors ≥ 16 was performed using the standard addition method. Compounds were quantified using GC–MS (7890B-5977, Agilent Technologies Inc., Santa Clara, CA, USA) in the selected ion mode (SIM). Standard solutions of the key odor compounds (7 different concentrations) were injected into the draft beer along with internal standards. The concentration method was the same as described in 3.4. Ethyl 2-ethylbutyrate was used as an internal standard for ester compounds, and 2-methyl-3-heptanone was used as an internal standard for the remaining compounds. The concentrations and specific amounts of the two internal standard solutions used in the analysis were ethyl 2-ethylbutyrate (0.876 mg/mL, 1 μL) and 2-methyl-3-heptanone (0.816 mg/mL, 10 μL). A standard curve was made using the ratio of the ion peak area of the quantified compound after the addition of the standard to the ion peak area of the internal standard minus the original ratio in the sample and the added concentration of each compound. In this case, the former were the vertical coordinates, and the latter were the horizontal coordinates. We calculated the concentration of the corresponding compound using each standard curve. The results of three parallel tests were averaged and used to determine the concentration of the quantitative compounds.

An important metric for evaluating the intensity of a compound’s odor in a sample is the OAV. According to related studies [40] and the alcohol content of the beer samples in this study, the OAV was determined by dividing its concentration by the odor threshold in an ethanol solution of approximately 3%.

### 3.8. Sensory Evaluation

Sensory assessments were performed by 25 trained graduate students (aged 21–25, 10 males and 15 females) from the Molecular Sensory Science Laboratory of Beijing Technology and Business University (Beijing, China). Samples were evaluated using a descriptive sensory analysis method. Team members were trained following the approach explained by Zhang et al. to make sure that they could correctly identify the odor characteristics of the draft beer [41].

Based on the relevant study [22] and the results of our sensory team evaluation, the odor profile of draft beer was classified into six categories: fruity/sweet, floral, roasted/malty, raw green, alcohol-like, and unpleasant odors. Unpleasant odors included sour and some unacceptable odors. The team members evaluated specific odor attributes and reference standards for various odors, which were described as follows: isoamyl acetate (1.5 mg/L, fruity/sweet), phenylethyl alcohol (100 mg/L, floral), 3-methyl-1-butanol (16.8 mg/L, roasted/malty), acetaldehyde (10 mg/L, raw green), ethanol (15 g/L, alcohol-like), and acetic acid (200 mg/L, unpleasant, sour). To conduct the evaluations, each beer sample (20 mL) was poured into a 50 mL opaque cup. The four brands of beer were randomly assigned three-digit codes. Meanwhile, it was ensured that the temperature of the sensory environment was 25 °C. Each brand of beer was assessed three times.

The evaluator assessed the intensity of the odor of the beer using a score range of 0–9, where 0 represented the weakest odor, and 9 represented the strongest. A rating between 0 and 3 indicated an odor attribute that was either barely detectable or very faint. A rating of 4–6 denoted a noticeable odor characteristic, while a score of 7–9 indicated a strong odor characteristic. Following each evaluation, the panelists took a 5 min break to use coffee beans to relieve olfactory fatigue. The ultimate score of the sample was determined by averaging the scores from the three assessments.

### 3.9. Aroma Recombination

The compounds with OAV > 1 in the sample were mixed in an alcoholic solution at their respective quantitative concentrations. The ethanol concentration, as well as the pH of this solution, was kept the same as the original samples (Table 3). This process was used to create odor RS. Evaluators simultaneously scored the intensity of each odor characteristic of the RS with the OS to determine its similarity to the OS.

### 3.10. Statistical Analysis

A principal component analysis (PCA) was performed by SIMCA-P 14.1 (Umetrics, Umeå, Sweden). Data handling of the E-nose was carried out using WinMuster E-nose software version 1.6.2 (Airsense Analytics GmbH, Schwerin, Germany). The remaining charts were created using Microsoft Office Excel 2019 and Origin 2022 (Origin Lab Corporation, Northampton, MA, USA). One-way analysis of variance (ANOVA) and Duncan’s multiple range test were used to analyze the experimental data using SPSS Statistics 26 (IBM, New York, NY, USA) to determine whether there was a significant difference (*p* < 0.05). All experiments were performed in three parallels. The final results were expressed as the mean ± standard deviation (SD).

## 4. Conclusions

In this study, the relationship between the flavor properties of four draft beers and key aroma active substances was investigated by means of molecular sensory science. E-nose results revealed that the overall odors of the four draft beers could be differentiated from one another. Furthermore, a sensory evaluation showed that the overall odor characteristics of the draft beers were primarily characterized by fruity/sweet, floral, and roasted/malty odors. Overall, 55 odor-active compounds were detected using GC×GC–O–MS, and the application of AEDA and OAV analyses revealed 22 important odor compounds. Among these compounds, 11 key odor-active compounds had OAVs > one, including ethyl isovalerate, isoamyl acetate, isoamyl propionate, ethyl caproate, 2-phenylethyl acetate, 2-methyl-1-propanol, 1-butanol, 3-methyl-1-butanol, 1-hexanol, phenylethyl alcohol, and 3-hydroxy-2-butanone. These compounds significantly contributed to the general odor of the draft beer. The PCA analysis results showed that the key odor-active compounds could accurately characterize the flavor properties of the samples, and they were the key odor-active compounds that caused the difference in sensory evaluation of different brands of beer. Aroma recombination revealed that the RS closely matched the odor profile of a draft beer. This study offers valuable insights into the odor characteristics of mainstream draft beers in the Chinese market. Meanwhile, it preliminarily revealed the odor-active compounds responsible for the differences in the sensory evaluation of different brands of beers and provided a theoretical basis for objectively evaluating and improving the quality of different brands of draft beer.

## Figures and Tables

**Table 1:** Odor-active compounds in samples *^1^*.

No.	RI *^2^* (DB-WAX)	Compounds *^3^*	Aroma	FD Factor *^4^*							
				S1		S2		S3		S4	
				LLE	SPME	LLE	SPME	LLE	SPME	LLE	SPME
**Fruity/sweet odor**											
1	890	Ethyl acetate	fruity, pineapple-like	- *^5^*	1	-	2	-	1	-	1
2	954	Ethyl propionate	fruity	-	32	-	8	-	32	-	16
3	969	Propyl acetate	fruity	-	2	-	-	-	-	-	1
4	1001	Ethyl butyrate	fruity, pineapple-like	32	16	8	8	64	32	16	8
5	1005	Isobutyl acetate	fruity	-	1	-	1	-	1	-	1
6	1021	Butyl acetate	fruity, banana-like	2	-	-	-	-	-	4	2
7	1026	Ethyl isovalerate	fruity	4	16	-	8	-	8	1	16
8	1032	1-Propanol	fruity	-	1	-	1	-	-	-	4
9	1035	2-Methyl-1-propanol	fruity	512	2	128	2	256	2	512	1
10	1068	Isoamyl acetate	fruity, banana-like	256	128	128	32	64	32	128	64
11	1083	1-Butanol	fruity, apple-like	16	8	2	2	16	2	8	4
12	1184	Isoamyl propionate	fruity	-	8	-	16	-	32	-	8
13	1185	Ethyl caproate	fruity, pineapple-like	32	16	16	8	64	32	16	8
14	1188	3-Methylbutyl 2-methylpropanoa	fruity	-	-	-	1	-	-	-	-
15	1202	Ethyl pyruvate	fruity	4	-	1	-	-	-	1	-
16	1217	Amyl acetate	fruity	-	-	-	-	-	-	-	4
17	1273	Ethyl lactate	fruity	16	8	2	4	4	4	8	8
18	1321	Ethyl heptanoate	fruity	-	1	2	1	1	-	2	1
19	1325	1-Hexanol	fruity, hawthorn-like	32	4	32	16	64	32	8	-
20	1401	Ethyl caprylate	fruity	2	1	2	4	2	2	2	1
21	1462	Ethyl 3-hydroxybutyrate	fruity	1	-	-	-	-	-	-	-
22	1471	Propionic acid	fruity	1	-	2	-	-	-	-	-
23	1487	2,3-Butanediol	fruity	2	-	1	-	8	-	1	-
24	1505	Ethyl nonanoate	fruity, grape-like	8	-	1	-	-	-	8	-
25	1607	Ethyl caprate	fruity	4	16	1	8	4	32	4	16
26	1626	Diethyl succinate	fruity	-	-	-	-	-	-	1	-
27	879	2-Methylfuran	sweet	-	2	-	4	-	-	-	2
28	969	2,3-Butanedione	sweet	-	-	-	1	-	-	-	1
29	1225	1-Hydroxyacetone	sweet	1	-	1	-	-	-	1	-
30	1228	Hexyl acetate	sweet	2	-	2	-	2	-	-	-
31	1449	2-Ethylhexanol	sweet	-	-	2	-	4	-	-	-
32	1596	Furfuryl alcohol	sweet	64	16	32	4	32	8	8	4
33	1214	3-Hydroxy-2-butanone	creamy	2	4	16	8	1	2	16	4
34	1445	2-Acetylfuran	caramel-like	1	-	1	-	4	-	1	-
35	1573	gamma-Butyrolactone	caramel-like	2	-	1	-	4	-	2	-
**Floral odor**											
36	1164	1-Pentanol	flowery	2	-	2	-	4	4	2	-
37	1354	1-Heptyl acetate	flowery	64	16	8	2	32	8	8	4
38	1837	2-Phenylethyl acetate	flowery, rose-like	16	64	4	32	4	32	8	64
39	1958	Phenethyl alcohol	flowery, rose-like	64	32	64	32	16	8	128	64
**Roasted/malty odor**											
40	917	3-Methylbutyraldehyde	malty	-	4	-	2	-	-	-	4
41	1170	3-Methyl-1-butanol	roasted, malty	8	16	32	16	8	2	64	32
42	1397	Furfural	nutty-like	1	2	1	2	1	-	2	4
43	1004	2,3-Pentanedione	nutty-like	4	-	1	-	-	-	-	-
44	1469	Benzaldehyde	nutty-like	8	-	4	-	-	-	4	-
**Raw green odor**											
45	1053	Hexanal	fresh aroma	1	-	1	-	1	-	1	-
46	1447	Octyl acetate	fresh aroma	4	-	-	-	2	-	-	-
47	1471	Decanal	cucumber-like	8	-	8	-	32	-	16	-
**Alcohol-like odor**											
48	931	Ethanol	alcohol-like	-	1	-	1	-	1	-	1
49	1205	2-Methyl-1-butanol	alcohol-like	-	-	-	1	-	-	-	-
**Unpleasant odor**											
50	1372	Acetic acid	sour	64	-	32	-	8	-	64	-
51	1563	Butyric acid	sour	16	-	8	-	32	-	2	-
52	1861	Hexanoic acid	sour	16	16	4	8	8	4	16	16
53	1608	Isovaleric acid	unpleasant	64	-	16	-	32	-	32	-
54	1664	3-Methylthiopropanol	unpleasant	2	-	4	-	4	-	4	-
55	2092	Octanoic acid	unpleasant	4	-	8	-	8	1	2	-

*^1^* Draft beer of Snowflake (S1), Tsingtao (S2), Budweiser (S3) and Yanjing (S4). *^2^* RI, retention indices (DB-WAX: 30 m × 0.25 mm, 0.25 μm film thickness). *^3^* The compound was identified by comparing its RI, mass spectrum, and odor quality with the authentic compound by GC×GC–O–MS. *^4^* FD factor, flavor dilution factor. *^5^* “-” not detected.

**Table 3:** Basic information about the samples S1–S4.

Samples	Raw Materials	Wort Concentration (°P)	Alcohol Content (%vol)	Acidity (μmol/L)	pH	Age
S1	water, malt, rice, hop products	8.0	2.5	45.7	4.3	5 May 2021
S2	water, malt, rice, hop products	8.0	3.1	39.8	4.4	25 June 2021
S3	water, malt, rice, hop products, yeast	8.0	3.1	52.5	4.3	4 January 2023
S4	water, malt, rice, hop products	8.0	2.5	53.7	4.3	8 March 2021

## Data Availability

Data are contained within the article.

## Supplementary Materials

The following supporting information can be downloaded at: https://www.mdpi.com/article/10.3390/molecules29112537/s1, Table S1: Retention indices of odor-active compounds; Table S2: Classification and odor scores of odor-active compounds base on the results of aroma extraction dilution analysis (AEDA); Table S3: Sensory evaluation of aroma recombination models and four brands of draft beer.
