# Supplementary material for: Characterization of Key Odor-Active Compounds in Draft Beers for the Chinese Market Using Molecular Sensory Science Approaches
Source: Molecules. 2024 May 28;29(11):2537. doi: 10.3390/molecules29112537 (PMC11173443; doi:10.3390/molecules29112537)
Supplement: Supplementary file 1 [file molecules-29-02537-s001.zip › molecules-2948952-supplementary.pdf]

Table S1. Retention indices of odor-active compounds.

No.	Compounds	RI [1](DB-WAX)	RI [2]
		**Fruity/sweet odor**	
1	Ethyl acetate	890	884
2	Ethyl propionate	954	961
3	Propyl acetate	969	977
4	Ethyl butyrate	1001	1044
5	Isobutyl acetate	1005	1017
6	Butyl acetate	1021	1049
7	Ethyl isovalerate	1026	1056
8	1-Propanol	1032	1038
9	2-Methyl-1-propanol	1035	1090
10	Isoamyl acetate	1068	1112
11	1-Butanol	1083	1150
12	Isoamyl propionate	1184	1188
13	Ethyl caproate	1185	1220
14	3-Methylbutyl2-methylpropanoa	1188	1183
15	Ethyl pyruvate	1202	1234
16	Amyl acetate	1217	1175
17	Ethyl lactate	1273	1316
18	Ethyl heptanoate	1321	1310
19	1-Hexanol	1325	1360
20	Ethyl caprylate	1401	1420
21	Ethyl 3-hydroxybutyrate	1462	1505
22	Propionic acid	1471	1508
23	2,3-Butanediol	1487	1542
24	Ethyl nonanoate	1505	1541
25	Ethyl caprate	1607	1633
26	Diethyl succinate	1626	1667
27	2-Methylfuran	879	877
28	2,3-Butanedione	969	977
29	1-Hydroxyacetone	1225	1275
30	Hexyl acetate	1228	1265
31	2-Ethylhexanol	1449	1484
32	Furfuryl alcohol	1596	1659
33	3-Hydroxy-2-butanone	1214	1271
34	2-Acetylfuran	1445	1501
35	gamma-Butyrolactone	1573	1602
		**Floral odor**	
36	1-Pentanol	1164	1209
37	1-Heptyl acetate	1354	1374
38	2-Phenylethyl acetate	1837	1791
39	Phenethyl alcohol	1958	1914
		**Roasted/malty odor**	

40	3-Methylbutyraldehyde	917	912
41	3-Methyl-1-butanol	1170	1185
42	Furfural	1397	1460
43	2,3-Pentanedione	1004	1056
44	Benzaldehyde	1469	1520
	**Raw green odor**		
45	Hexanal	1053	1091
46	Octyl acetate	1447	1471
47	Decanal	1471	1515
	**Alcohol-like odor**		
48	Ethanol	931	911
49	2-Methyl-1-butanol	1205	1208
	**Unpleasant odor**		
50	Acetic acid	1372	1429
51	Butyric acid	1563	1628
52	Hexanoic acid	1861	1851
53	Isovaleric acid	1608	1647
54	3-Methylthiopropanol	1664	1715
55	Octanoic acid	2092	2067

[1] RI, retention indices (DB-WAX).

[2] RI values from website：https://webbook.nist.gov/chemistry/.

Table S2. Classification and odor scores of odor-active compounds base on the results of aroma extraction dilution analysis (AEDA).

Odor descriptors	Number of compounds in samples [1] (percentage [2], %) / Score[4] of draft beer sample (percentage [3], %)							
	S1		S2		S3		S4	
	LLE	SPME	LLE	SPME	LLE	SPME	LLE	SPME
Fruity/sweet	22 (56.4) / 996 (74.2)	20 (71.4) / 283 (65.2)	21 (56.8) / 381 (65.9)	21 (70.0) / 139 (59.1)	17 (53.1) / 594 (75.6)	16 (66.7) / 254 (80.9)	21 (58.3) / 748 (67.9)	22 (73.3) / 175 (48.1)
Floral	4(10.3) / 146 (10.9)	3(10.7) / 112 (25.8)	4 (10.8) / 78 (13.5)	3 (10.0) / 66 (28.1)	4 (12.5) / 56 (7.1)	4 (16.7) / 52 (16.6)	4 (11.1) / 146 (13.3)	3 (10.0) / 132 (36.3)
Roasted/malty	4 (10.3) / 21 (1.6)	3 (10.7) / 22 (5.1)	4 (10.8) / 38 (6.6)	3 (10.0) / 20 (8.5)	2 (6.3) / 9 (1.1)	1 (4.2) / 2 (0.6)	3 (8.3) / 70 (6.4)	3 (10.0) / 40 (11.0)
Raw green	3 (7.7) / 13 (1.0)	0 (0.0) / 0 (0.0)	2 (5.4) / 9 (1.6)	0 (0.0) / 0 (0.0)	3 (9.4) / 35 (4.5)	0 (0.0) / 0 (0.0)	2 (5.6) / 17 (1.5)	0 (0.0) / 0 (0.0)
Alcohol-like	0 (0.0) / 0 (0.0)	1 (3.6) / 1 (0.2)	0 (0.0) / 0 (0.0)	2 (6.7) / 2 (0.9)	0 (0.0) / 0 (0.0)	1 (4.2) / 1 (0.3)	0 (0.0) / 0 (0.0)	1 (3.3) / 1 (0.3)
Unpleasant	6 (15.4) / 166 (12.4)	1 (3.6) / 16 (3.7)	6 (16.2) / 72 (12.5)	1 (3.3) / 8 (3.4)	6 (18.8) / 92 (11.7)	2 (8.3) / 5 (1.6)	6 (16.7) / 120 (10.9)	1 (3.3) / 16 (4.4)

Total	39 / 1342	28 / 434	37 / 578	30 / 235	32 / 786	24 / 314	36 / 1101	30 / 364

[1] Draft beer of Snowflake (S1), Tsingtao (S2), Budweiser (S3) and Yanjing (S4).

[2, (3)] The number (score) of each odor descriptor compound accounts for the percentage of the total number (score) of odor-active compound.

[4] The sum of FD factor of all compounds in each odor attribute was used as its intensity score.

Table S3. Sensory evaluation of aroma recombination models and four brands of draft beer.

	Sensory score (± SD [1])							
**Odor qualities [2]**	**S1**		**S2**		**S3**		**S4**	
	**OS [3]**	**RS [4]**	**OS**	**RS**	**OS**	**RS**	**OS**	**RS**
Fruity/sweet	6.73 ± 1.01	6.64 ± 1.03	4.64 ± 1.29	5.45 ± 1.04	4.00 ± 1.79	3.82 ± 0.87	5.36 ± 1.03	5.27 ± 1.10
Floral	5.27 ± 1.42	5.82 ± 0.75	4.55 ± 1.86	5.09 ± 0.94	3.91 ± 1.64	3.36 ± 1.12	6.09 ± 1.45	5.36 ± 1.69
Roasted/malty	5.09 ± 2.26	4.09 ± 1.30	4.36 ± 1.29	3.55 ± 1.29	3.91 ± 1.14	3.45 ± 1.13	6.27 ± 1.19	5.18 ± 1.47
Raw green	3.00 ± 2.00	3.64 ± 1.12	4.09 ± 1.30	3.82 ± 1.17	4.82 ± 2.14	4.91 ± 1.14	4.36 ± 1.91	3.91 ± 1.38
Alcohol-like	5.00 ± 1.61	4.82 ± 1.78	5.91 ± 1.58	5.64 ± 1.57	4.55 ± 1.75	4.36 ± 1.21	4.36 ± 2.16	4.27 ± 1.49
Unpleasant	2.82 ± 1.83	2.73 ± 0.90	2.09 ± 0.83	2.36 ± 1.21	1.91 ± 0.94	2.18 ± 1.17	3.09 ± 1.51	3.36 ± 1.43

[1] SD, standard deviation.

[2] Odor quality which used for evaluate the aroma characteristics of four brands of draft beer.

[3] OS: Draft beer of S1-S4.

[4] RS was prepared in an aqueous alcohol solution that represents the ethanol concentration and pH of the original beer with adding aroma compounds (OAVs ＞1) at their concentration in four brands of draft beer.
